# Two-year results after combined phacoemulsification and iris-fixated phakic intraocular lens removal

**DOI:** 10.1007/s00417-021-05442-3

**Published:** 2021-10-16

**Authors:** Zoraida Solaiga Gaurisankar, Gwyneth A. van Rijn, Yanny Y. Y. Cheng, Gregorius P. M. Luyten, Jan-Willem M. Beenakker

**Affiliations:** 1 Department of Ophthalmology, Leiden University Medical Center, Albinusdreef 2, 2333 ZA Leiden, The Netherlands; 2 Department of Radiology, C.J. Gorter Center for High-Field MRI, Leiden University Medical Center, Leiden, The Netherlands

**Keywords:** pIOL explantation, pIOL removal, Surgical technique, Iris-claw lens, Iris-fixated pIOL

## Abstract

**Purpose:**

To describe and present results after a technique for cataract surgery combined with explantation of an iris-fixated phakic intraocular lens (IF-pIOL).

**Methods:**

The medical records of all patients, who had undergone cataract surgery combined with IF-pIOL explantation and subsequent implantation of a posterior chamber IOL by the Single Incision Technique (SIT), were reviewed. Data collection included preoperative and postoperative corrected distance visual acuity (CDVA), manifest refraction, and endothelial cell density (ECD) up to a follow-up time of 24 months.

**Results:**

Fifty myopic eyes (34 patients) and 9 hyperopic eyes (6 patients) had undergone a SIT procedure mainly because of cataract (67%). Postoperative CDVA improved in both the myopic eyes to 0.16 ± 0.37 logMAR, as in the hyperopic eyes to − 0.10 ± 0.55 logMAR with no eyes having loss of Snellen lines. Mean postoperative spherical equivalent was − 0.34 ± 0.72 D and − 0.10 ± 0.55 D, respectively. ECD loss 6 months after surgery was 5% and remained stable thereafter.

**Conclusion:**

SIT for combined phacoemulsification and IF-pIOL removal yields good visual and refractive results and is a safe procedure in regard to ECD loss. The technique has advantages over the conventional procedure and is easy to perform.

**Supplementary Information:**

The online version contains supplementary material available at 10.1007/s00417-021-05442-3.


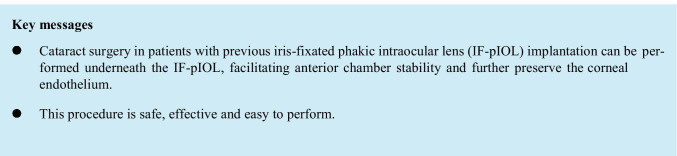


## Introduction

The implantation of a phakic intraocular lens (pIOL) allows treatment of (high) refractive errors, with the advantage of sparing the crystalline lens. One of the most common anterior chamber pIOLs is the iris-fixated (IF) Artisan pIOL [1] and has been demonstrated to be an effective, predictable, and stable procedure for all models [1–3]. However, regular lifetime follow-up is needed, as increased endothelial cell density (ECD) loss remains a concern after any type of anterior chamber pIOL. Different studies have demonstrated ECD loss to be the most important risk factor in patients with an IF-pIOL [4–8]. Excessive ECD loss and cataract formation are the main reasons for explantation of IF-pIOL. Explantation of the pIOL is then combined with phacoemulsification and placement of a posterior IOL [9, 10]. This procedure carries the risk of additional ECD loss due to the phacoemulsification [11, 12] and manipulation of the pIOL in the anterior chamber.

Most surgeons will first remove the IF-pIOL and sequentially perform the phacoemulsification through a separate incision, inserting a posterior chamber IOL in the capsular bag at the end [13, 14]. Khokhar et al. [15] recently described an alternative surgical approach, which is already applied in our clinic since 2000. This technique consists of performing phacoemulsification underneath the pIOL through a main corneoscleral incision. The same incision is then further opened to remove the IF-pIOL as a last step before placing the posterior chamber IOL in the capsular bag. Using the latter technique, it is thought that the pIOL shields for ECD damage during cataract surgery and the anterior chamber are better maintained with less risk for iris prolapse during phacoemulsification.

In this study, we describe the surgical technique of performing cataract surgery underneath the pIOL in patients, previously treated with an (toric) Artisan or Artiflex (Ophtec BV) IF-pIOL and we present the safety and visual and refractive outcomes of this procedure.

## Methods

This retrospective case study adhered to the tenets of the Declaration of Helsinki and was approved by the medical ethical committee of the Leiden University Medical Center (LUMC). All eligible patients signed an informed consent. Medical records from our clinics were reviewed of all patients with a history of IF-pIOL implantation for refractive correction of myopia or hyperopia between 2000 and 2019 and who had undergone the Single Incision Technique for combined phacoemulsification, pIOL explantation, and IOL implantation (hereafter referred as to “SIT”) during follow-up. All SIT surgeries have been performed by an experienced surgeon (GL/YC) at the LUMC, Leiden. The pIOL used for refractive correction included the Artisan Myopia pIOL model 204 or 206, Artisan Hyperopia pIOL model 203, Artisan toric pIOL, and Artiflex myopia pIOL. Calculation of posterior chamber IOL power was performed with the SRK/T formula [16], with the exception of short eyes (22.0 mm or shorter), for which the Holladay 2 formula was used [17]. The IOL model chosen for implantation depended on the availability and the surgeon’s preference and included Tecnis ZCB00, PCB00, or ZA9003, and Sensar AR40 (Johnson&Johnson); AcrySof MA60MA and SA60AT (Alcon Laboratories); Bigbag (Carl Zeiss Meditec AG).

### Preoperative evaluation

A detailed medical history was reviewed including patient’s age at the time of the pIOL implantation and at the time of the SIT procedure, the type and power of pIOL implant, the indication for phacoemulsification, and the type and power of posterior chamber IOL power implanted. Preoperative ocular examination included corrected distance visual acuity (CDVA) determined using Snellen charts, manifest refraction, and ECD measured by Topcon SP-2000P or Topcon SP-3000P noncontact specular microscope (Topcon Corporation). Data recorded on ECD included the ECD count (1) preoperative to pIOL implantation and (2) preoperative to the SIT and (3) postoperative to the SIT procedure. Preoperative axial length measurement was obtained with the Lenstar LS 900 (Haag-Streit AG) or IOLMaster (Carl Zeiss Meditec).

### Surgical technique

Video 1 shows the surgical procedure. After the pupil was fully dilated, the patient was prepped and draped. A main 3.0-mm limbal incision and 2 clear corneal side ports were created. The main incision was attempted to place at the steep axis to minimize postoperative astigmatism. The ophthalmic viscosurgical device (OVD) (Healon, Johnson & Johnson Vision Surgical) was injected into the anterior chamber to separate the pIOL from the crystalline lens and a continuous curvilinear capsulorhexis was created using forceps, followed by hydrodissection, phacoemulsification, and combined irrigation/aspiration (I/A). The OVD was then injected in the capsular bag, and anterior chamber. The main incision was then enlarged to 6.0 mm (except in the case of the Artiflex) and the pIOL was removed after de-enclavation of the haptics with the Budo forceps and disposable enclavation needle (Ophtec BV). Once the pIOL was removed, the posterior IOL was implanted in the capsular bag followed by closure of the main incision with one running or multiple intermittent 10–0 nylon sutures. Intraocular OVD was removed and the wounds were checked for closure. At the end of the surgery, intracameral cefuroxime and parabulbar betamethasone was administered. All surgeries were performed under either general or local anesthesia.

### Postoperative management

Follow-up examinations were typically scheduled at 1 month, 3 months, 6 months, 1 year, and 2 years. Postoperative examinations included CDVA and manifest refraction. Within the first 3 months, sutures were removed in case of residual corneal astigmatism. Postoperative ECD count was recorded at two follow-up points: within 6 months or between 6 and 24 months, to differentiate between ECD loss due to surgical trauma and ECD loss thereafter. For comparison of the ECD counts over time, we applied the recently proposed method, described by van Rijn et al. [18], to correct for systematic differences as result of the use of these different microscopes,

### Statistical analysis

Data was analyzed with IBM SPSS Statistics version 25 for Windows (SPSS Inc., Chicago, IL, USA). Descriptive statistics were generated: quantitative variables were expressed in means and standard deviations; qualitative variables were expressed as percentages and proportions of the total number of cases. Histograms and line diagrams were used to visualize data.

For visual and refractive outcomes, myopic and hyperopic results were listed separately and data recorded at the last follow-up was used as postoperative value for comparative analysis. Decimal CDVA values were converted to logarithm of minimum angle of resolution (logMAR) notation for calculations. We used paired Student’s *t* test to compare preoperative and postoperative visual acuity and refraction.

EC change was defined as the difference between the preoperative and postoperative examination and expressed as a percentage of the preoperative cell density. For analysis, a distinction is made between 2 groups: (1) eyes with low preoperative ECD (1000 cells/mm^2^ or less) and (2) eyes with a preoperative ECD of above 1000 cells/mm^2^. One-way analysis of variance (ANOVA) was used for overall comparison of the pre- and two postoperative ECD counts and post hoc comparisons were done with the Tukey test.

A *p*-value of < 0.05 was considered statistically significant.

## Results

### Patient characteristics

SIT was performed in 59 eyes of 40 patients of which 50 myopic eyes (34 patients) and 9 hyperopic eyes (6 patients). Mean axial length was 29.1 ± 2.3 mm and 21.4 ± 0.6 mm, respectively. The age at time of the procedure was 56.1 ± 14.1 years, after having the pIOL in situ for 145 ± 60 months. Mean ECD count preoperative to pIOL implantation was 2644 ± 412 in the myopic eyes and 2834 ± 502 in the hyperopic eyes. Independent sample T-test showed no significant difference between these two groups (*p* = 0.052). Of the myopic eyes, 2 eyes had retinal detachment surgery during follow-up between pIOL implantation and cataract surgery. Overall, cataract was the main reason for the SIT procedure in 42 eyes (71%), followed by EC loss in 17 eyes (29%). In the hyperopic eyes, EC loss was the main reason (67%) for pIOL explantation and cataract extraction. The implanted spherical pIOL power was − 12.2 ± 4.2 diopters (D) in the myopic and + 7.6 ± 1.6 D in the hyperopic eyes. In 8 out of 50 myopic eyes and 2 out of 9 hyperopic eyes, a toric Artisan was implanted and in 4 myopic eyes, an Artiflex was implanted. The rest of the eyes were implanted with an Artisan lens model 203, 204, or 206. Target refraction for the posterior IOL was emmetropia, except for 4 myopic eyes. These patients had chosen a target refraction of − 2.0 D. To reach target refraction, 4 myopic eyes had received a toric IOL; the remainder received a monofocal lens.

### Visual acuity and refraction

Table 1 shows the preoperative and postoperative clinical features of the study eyes at postoperative pIOL implantation and pre- and postoperative SIT.

**Table 1:** Visual acuity and refractive results preoperative and postoperative Single Incision Technique

Parameter	Postoperative pIOL	Preoperative SIT	Postoperative SIT
Myopic eyes *N* = 50	Mean time to SIT = 140 ± 62 months	Mean time to SIT = 5 ± 7 months	Mean time from SIT = 14 ± 9 months
Mean CDVA (logMAR)	0.08 ± 0.16	0.23 ± 0.40	0.09 ± 0.39*
Mean MRSE (D)	− 0.59 ± 0.92	− 1.62 ± 1.84	− 0.34 ± 0.72**
Mean deviation SE from target refraction (D)			− 0.08 ± 0.57
SE refraction within ± 0.5 D of intended (%)			72
SE refraction within ± 1.0 D of intended (%)			94
Hyperopic eyes *N* = 9	Mean time to SIT = 172 ± 45 months	Mean time to SIT = 7 ± 5 months	Mean time from SIT = 18 ± 10 months
CDVA (logMAR)	0.07 ± 0.11	0.12 ± 0.18	− 0.02 ± 0.11*
MRSE (D)	− 0.03 ± 0.64	− 0.59 ± 1.77	− 0.10 ± 0.55
Mean deviation SE from target refraction (D)			− 0.23 ± 0.34
SE refraction within ± 0.5 D of intended (%)			89
SE refraction within ± 1.0 D of intended (%)			100

*CDVA*, corrected distance visual acuity; *D*, diopters; *logMAR*, logarithm of minimum angle of resolution; *MRSE*, manifest refraction spherical equivalent; *pIOL*, phakic intraocular lens; *SE*, spherical equivalent; *SIT*, Single Incision Technique

^*^p-value < 0.05, paired samples *t*-test pre- and postoperative SIT

^**^p-value < 0.001, paired samples *t*-test pre- and postoperative SIT

Preoperative to the SIT procedure, both groups showed an overall myopization and improved CDVA.

**Fig. 1:**
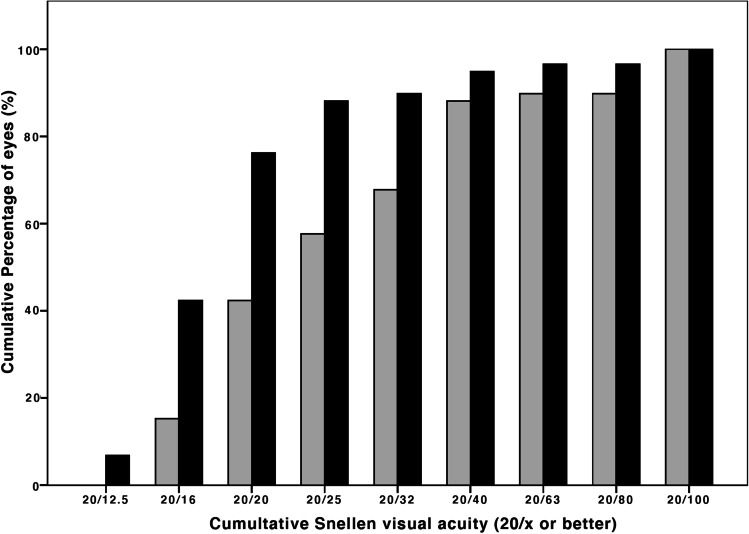
Preoperative (*gray bars*) and postoperative (*black bars*) corrected distance visual acuity (CDVA) after the Single Incision Technique of all eyes (*N* = 59) showing an overall gain in postoperative CDVA

**Fig. 2:**
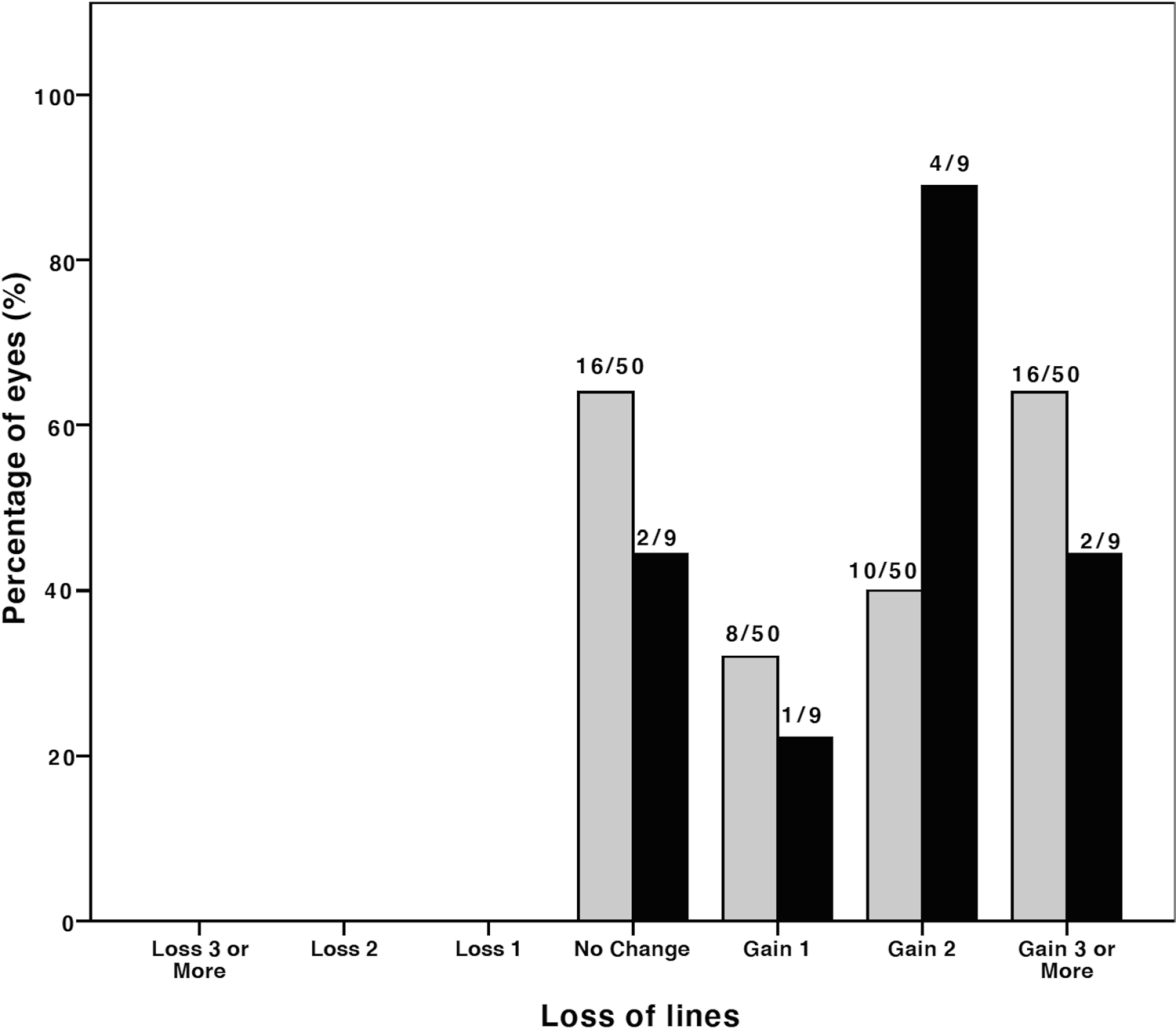
Difference between preoperative and postoperative corrected distance visual acuity after the Single Incision Technique (*N* = 59) for myopic (*gray bars*) and hyperopic (*black bars*) eyes. No eyes showed loss of Snellen visual acuity lines

**Fig. 3:**
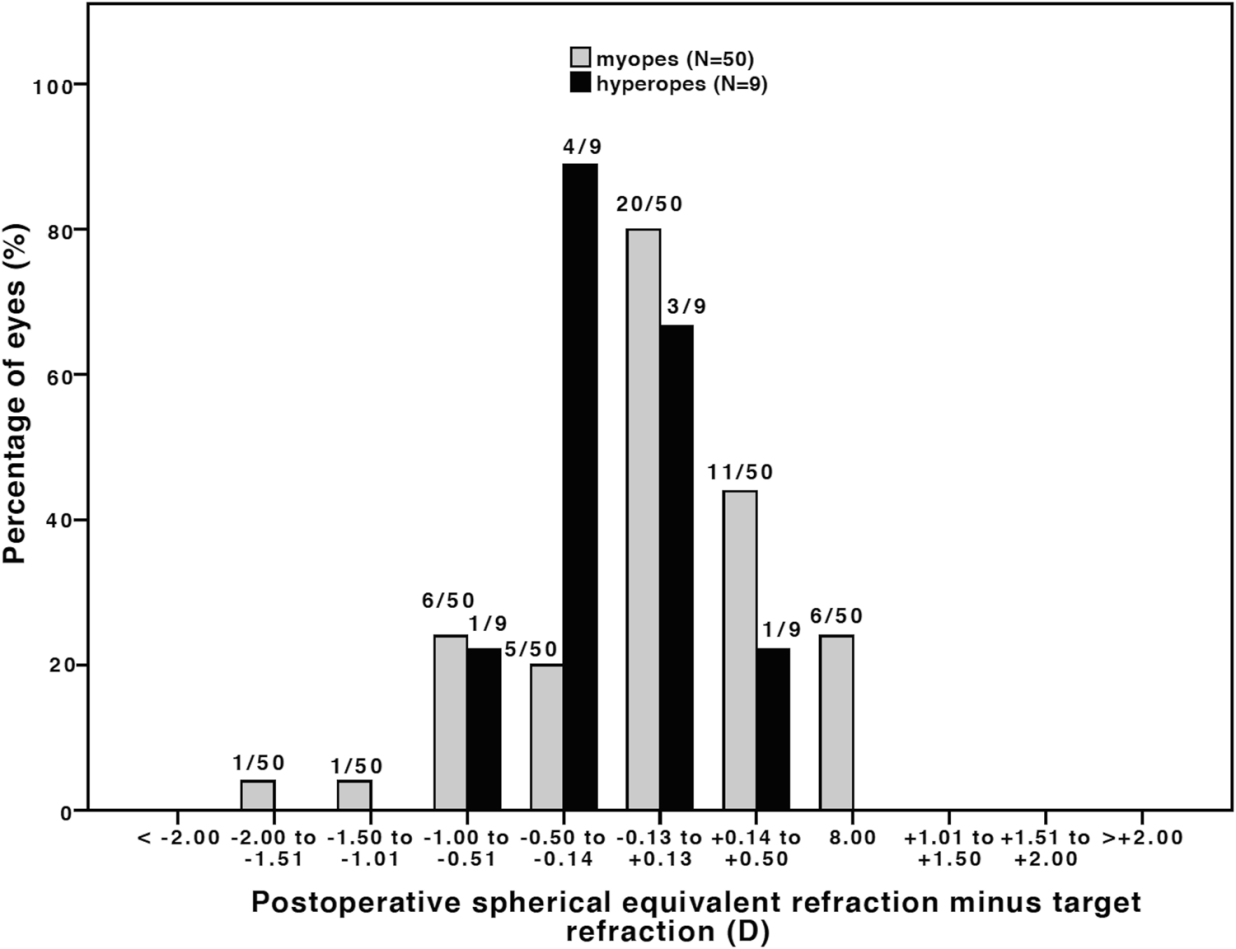
Spherical equivalent (SE) refractive accuracy after the Single Incision Technique (*N* = 59) for all myopic (*gray bars*) and hyperopic (*black bars*) eyes. 94% of the myopic eyes and 100% of the hyperopic eyes reached SE refraction within ± 1.0 D of intended

**Fig. 4:**
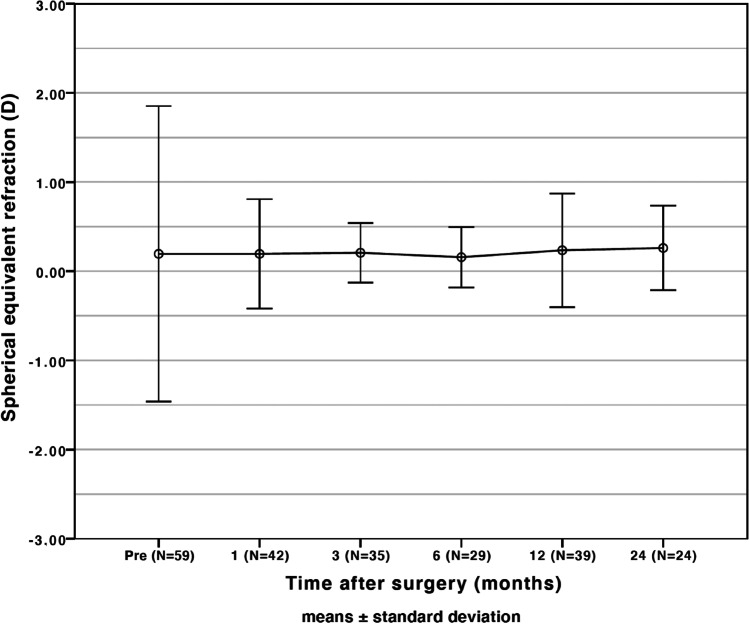
Stability of spherical equivalent refraction of all eyes (*N* = 59) showing stable postoperative refraction after the Single Incision Technique

Compared to preoperative results, the mean difference in CDVA in the myopic group was 0.16 ± 0.37 logMAR (*p* = 0.003) and − 0.05 ± 0.11 logMAR (*p* = 0.210) in the hyperopic group (Fig. 1). No eyes showed visual acuity loss of Snellen lines (Fig. 2). A satisfactory mean manifest refraction spherical equivalent (MRSE) of − 0.34 ± 0.72 logMAR and − 0.10 ± 0.55 logMAR was achieved in myopic and hyperopic eyes, respectively. The MRSE was less myopic postoperatively, in both groups: − 0.34 ± 0.72 D (*p* < 0.001) and − 0.10 ± 0.55 D (*p* = 0.385), respectively (Figs. 3 and 4). Postoperative refractive cylinder was within ≤ 0.50 D in 24/50 eyes (48%) and ≤ 1.00 D in 33/50 eyes (66%), compared to 27% and 56% preoperatively.

### Endothelial cell density

**Fig. 5:**
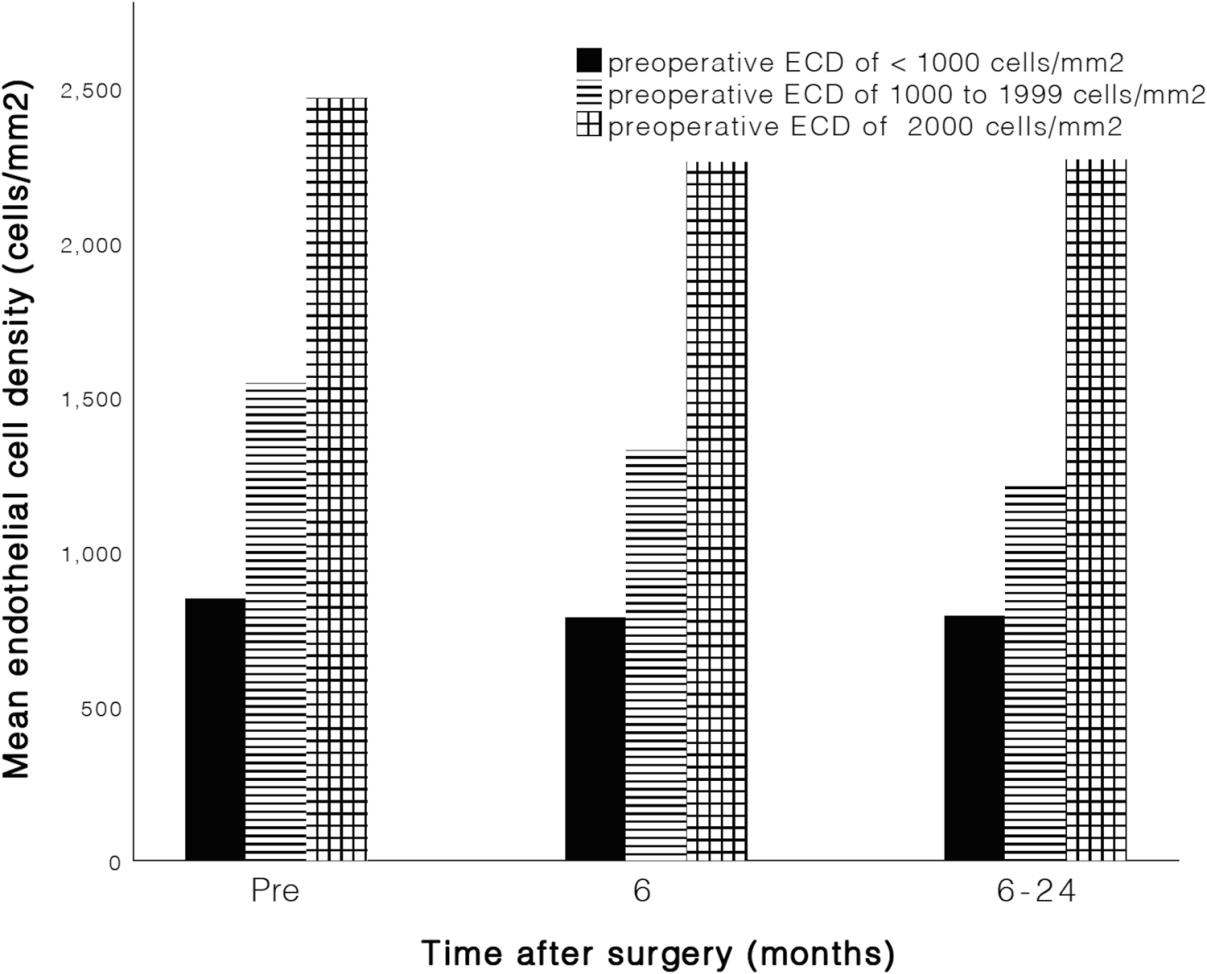
Bar graph of preoperative and postoperative endothelial cell density (ECD)

Overall postoperative ECD loss was − 5.4 ± 11.8% after 6 months and − 9.4 ± 17.0% after 6–24 months, compared to preoperative ECD. For a more detailed analysis of the ECD loss, a distinction is made between eyes with (1) a low preoperative ECD (less than 1000 cells/mm^2^), (2) a preoperative ECD of 1000 to 1999 cells/mm^2^, and (3) a preoperative ECD of 2000 or more cells/mm^2^, as seen in Table 2. ECD loss developed within the first 6 months postoperative, to be interpreted as a result of surgical trauma, was − 4.7 ± 12.0% in the first group − 4.0 ± 17.6% in the second group, and − 3.5 ± 7.3% in the third group. ECD loss developed 6 to 24 months postoperative was − 0.8 ± 23.8%, − 16.8 ± 22.7%, and − 7.7 ± 6.5%, respectively (Fig. 5). Using one-way ANOVA, there was no significant difference between the preoperative and postoperative ECD counts (*p* = 0.100).

**Table 2:** Endothelial cell results preoperative and postoperative Single Incision Technique

Parameter	Preoperative	Postoperative (6 months)	Postoperative (6–24 months)
ECD < 1000 cells/mm^2^	*N* = 8	*N* = 6	*N* = 6
Time interval to SIT (months)	− 6 ± 4	3 ± 2	14 ± 5
ECD (cells/mm^2^)	847 ± 148	785 ± 148	791 ± 138
ECD loss (%)		− 8.3 ± 10.8	− 0.8 ± 23.8
ECD 1000 to 1999 cells/mm^2^	*N* = 21	*N* = 11	*N* = 11
Time interval to SIT (months)	− 7 ± 11	3 ± 2	15 ± 7
ECD (cells/mm^2^)	1543 ± 355	1326 ± 385	1226 ± 339
ECD loss (%)		− 4.0 ± 17.6	− 16.8 ± 22.7
ECD ≥ 2000 cells/mm^2^	*N* = 30	*N* = 13	*N* = 18
Time interval to SIT (months)	− 11 ± 14	4 ± 4	22 ± 10
ECD (cells/mm^2^)	2466 ± 334	2269 ± 301	2260 ± 244
ECD loss (%)		− 3.5 ± 7.3	− 7.7 ± 6.5

*ECD*, endothelial cell density; *SIT*, Single Incision Technique

### Safety

The postoperative spherical equivalent of one eye (2%) deviated − 1.78 D from target refraction. This concerned a patient with keratoconus after toric IF-pIOL implantation. At time of the SIT, a monofocal IOL was placed. Because of this unsatisfactory refractive outcome, patient received an additional toric IF-pIOL 3 months after SIT, with good visual and refractive outcome.

Cataract surgery was complicated by a posterior capsular rupture in three eyes (5%) of which two eyes with vitreous loss. One myopic patient presented with a rhegmatogenous retinal detachment in one eye (2%) within 2 years after the SIT procedure.

## Discussion

In this paper, we describe an alternative surgical approach, the SIT, for cataract removal in patients with an (toric) IF-pIOL in situ for myopia or hyperopia. We evaluated in 59 eyes the efficacy and safety including the course of EC loss of this technique during a follow-up of 2 years. All eyes had a stable or gain in CDVA post-SIT, and no eyes had a loss of Snellen lines. The postoperative MRSE was stable during follow-up and was within ± 1.00 D of intended refraction in 94.0% in the myopic and 100.0% in the hyperopic group. We found an acceptable ECD loss of less than 10% 6 months postoperative.

The main reasons for explantation of IF-pIOL in our study were formation of visually significant age-related cataract in myopic and ECD loss in hyperopic eyes. These findings are in line with previous literature [10, 19, 20]. Pigment dispersion has been reported as a complication of Artisan pIOL [21, 22] and was present in one hyperopic eye but was not the reason for the SIT procedure. The results of removal of IF-pIOL, combined with phacoemulsification, have been described in a study [13] by de Vries et al. who report a comparable effect in 36 eyes on CDVA and postoperative SE using the conventional surgical technique. That study found a smaller rate of ECD loss at 6 months of 3.5 ± 13.2 cells/mm^2^. However, the endothelial damage after routine cataract surgery in “virgin” eyes is similar to our findings [23, 24]. Comparable results on CDVA and postoperative SE are described in a more recent study by Vargas et al. [25] including 43 eyes. In this study, the pIOL is removed through a scleral incision which was sutured before performing phacoemulsification through a 2.8-mm clear corneal incision. This study found significant postoperative ECD loss compared to preoperative of 20.7% (*p* = 0.002). The larger amount of ECD loss in this study compared to our findings might be the result of a lower mean preoperative ECD mean ECD of 1408 cells/mm^2^ compared to our study (1918 cells/mm^2^). In our study, we discuss the results of a combined procedure of pIOL explantation and phacoemulsification. However, it is worth noticing that alternatively the pIOL explantation and phacoemulsification can also be performed in two individual sequential procedures. The advantages of this method are that it is less complex and phacoemulsification can be performed using sutureless incisions. The disadvantage is that it is more time-consuming and more burdensome for the patient.

To our knowledge, this retrospective study is the first to evaluate results of the SIT for combined phacoemulsification at which cataract is removed while the pIOL is still in situ. The procedure is easy to perform and has some advantages [15] over the conventional method. First of all, by performing the phacoemulsification through a 2.2‑mm incision, anterior chamber stability is well controlled. Secondly, the OVD above and beneath the IF-pIOL protects the cornea endothelium during phacoemulsification.

Nevertheless, EC damage due to surgical trauma remains an important parameter for this procedure. Our results yielded an acceptable ECD loss due to surgical trauma, but some cases show unreal gains (and drops) in ECD as the result of measurement error. The reliability of EC analysis is a well-discussed topic [26–29] with count errors of up to 9% with the SP2000P [29]. In addition, in our study, both the Topcon SP-2000P as the SP-3000P specular microscope was used during follow-up. We therefore applied the recently proposed method, described by van Rijn et al. [18], to correct for systematic differences as result of the use of these different microscopes.

It should further be noted that a bigger sample size and a prospective study design would improve the strength of our findings. Typically, patients missed some of the follow-up visits. To still optimally analyze the available data, data of the last available postoperative follow-up visit was used for comparison. Furthermore, it is noteworthy that patients with pIOL having cataract surgery followed by pars plana vitrectomy at the same day due to retinal detachment were not included in this analysis as the retinal surgeons did not use the described SIT procedure.

In conclusion, it can be stated that phacoemulsification beneath the IF-pIOL is an effective and safe procedure as regards for patients in need of IF-pIOL removal. Good visual outcomes, predictable refractive outcomes, and acceptable ECD loss at 6 months of less than 10% are achieved. The technique is easy to perform and has the advantages over conventional combined surgery that the pIOL functions as a protective shield for the endothelium and the anterior chamber are better maintained.

## Supplementary Information

Below is the link to the electronic supplementary material.

Supplementary file1 (MPG 15518 KB) Video 1 Video demonstrating the surgical procedure of the Single Incision Technique for combined phacoemulsification and iris-fixated phakic intraocular lens explantation

## Data Availability

The data that support the findings of this study are available from the corresponding author, ZSG, upon reasonable request.

## Code availability

The data code (SPSS Syntax) that support the findings of this study are available from the corresponding author, ZSG, upon reasonable request.
